# Current desires of conspecific observers affect cache-protection strategies in California scrub-jays and Eurasian jays

**DOI:** 10.1016/j.cub.2016.11.020

**Published:** 2017-01-23

**Authors:** Ljerka Ostojić, Edward W. Legg, Katharina F. Brecht, Florian Lange, Chantal Deininger, Michael Mendl, Nicola S. Clayton

**Affiliations:** 1 Department of Psychology, University of Cambridge, Downing Street, Cambridge CB2 3EB, UK; 2 Department of Psychology, University of Tübingen, Schleichstrasse 4, 72076 Tübingen, Germany; 3 Department of Psychology, Technische Universität Braunschweig, Pockelsstrasse 14, 38106 Braunschweig, Germany; 4 Centre for Behavioural Biology, School of Veterinary Science, University of Bristol, Langford House, Langford, Bristol BS40 5DU, UK

## Abstract

Many corvid species accurately remember the locations where they have seen others cache food, allowing them to pilfer these caches efficiently once the cachers have left the scene [1]. To protect their caches, corvids employ a suite of different cache-protection strategies that limit the observers’ visual or acoustic access to the cache site 2, 3. In cases where an observer’s sensory access cannot be reduced it has been suggested that cachers might be able to minimise the risk of pilfering if they avoid caching food the observer is most motivated to pilfer [4]. In the wild, corvids have been reported to pilfer others’ caches as soon as possible after the caching event [5], such that the cacher might benefit from adjusting its caching behaviour according to the observer’s current desire. In the current study, observers pilfered according to their current desire: they preferentially pilfered food that they were not sated on. Cachers adjusted their caching behaviour accordingly: they protected their caches by selectively caching food that observers were not motivated to pilfer. The same cache-protection behaviour was found when cachers could not see on which food the observers were sated. Thus, the cachers’ ability to respond to the observer’s desire might have been driven by the observer’s behaviour at the time of caching.

## Teaser

Ostojić *et al.* report that corvids employ cache-protection strategies that are sensitive to the current motivational state of an observing conspecific. This cache-protection strategy seems to be a response to the observer’s overt behavior, thus suggesting that cache-protection strategies need not be based on complex cognitive abilities like mentalizing.

## Main Text

California scrub-jays (*Aphelocoma californica*) and Eurasian jays (*Garrulus glandarius*) served as model species for the current study. Both species are known to engage in a variety of cache-protection strategies 1, 2, 3, 4, 6, 7 and both have previously been shown to be able to disengage from their current desire in order to cache food they will desire at the time they will retrieve their caches 8, 9. Thus, both species would appear to have the pre-requisites necessary to employ a cache-protection strategy that is sensitive to another’s desire.

**Figure 1:**
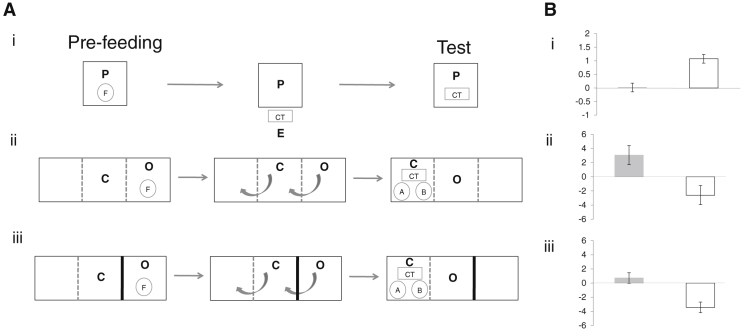
Experimental set-up and results. (A) Experimental set-up in the (i) pilfering experiment and the (ii) seen and (iii) unseen conditions of the caching experiment. The black squares denote the testing cages/compartments with mesh sides. In the (i) pilfering experiment, the pilferer P was pre-fed one of the three different foods F (either maintenance diet, food A or food B) for 15 minutes. Subsequently, the pilferer could observe the experimenter E cache 8 pieces of food A and food B into the caching tray CT positioned just outside the testing cage/compartment. In the test, the caching tray was positioned inside the pilferer’s cage/compartment and the bird was given 15 minutes time to pilfer the caches. In the (ii) seen condition of the caching experiment, cacher C could see (grey dashed line between cages/compartments) the observer O being pre-fed different foods (maintenance diet, food A or food B) for 15 minutes. Subsequently, the cacher and observer moved compartments as indicated by the grey arrows. In the test, the cacher could cache 50 pieces of food A and 50 pieces of food B into the caching tray for 15 minutes in sight of the observer (grey dashed line). In the (iii) unseen condition of the caching experiment, the observer was pre-fed different foods (either maintenance diet, food A or food B) for 15 minutes out of sight of the cacher (black solid line between cages/compartments). Again, the cacher and observer moved compartments (grey arrows). In the test the cacher could cache 50 pieces of food A and 50 pieces of food B into the caching tray for 15 minutes in sight of the observer (grey dashed line). (B) Mean difference in the number of pieces of food A minus number of pieces of food B (i) pilfered when the jays were pre-fed food A (grey bars) and when the jays were pre-fed food B (white bars) and cached in the (ii) seen and (iii) unseen conditions when the observer was pre-fed food A (grey bars) and when the observer was pre-fed food B (white bars). The performance of California scrub-jays and Eurasian jays did not differ in any of the experiments, such that data from both species were pooled for all analyses and graphs (pilfering experiment: total n = 16; 10 California scrub-jays and 6 Eurasian jays; caching experiment: total n = 16, 9 California scrub-jays and 7 Eurasian jays). Values under zero denote a decrease in the preference for food A over food B relative to the baseline (pre-fed maintenance diet) and values over zero denote an increase in the preference for food A over B relative to the baseline. Error bars denote the standard error of the mean.

A cache-protection strategy sensitive to an observer’s desire is only beneficial if the observer’s current desire influences its pilfering behaviour. Thus, in a pilfering experiment we manipulated the jays’ desire by pre-feeding them a particular food to induce a decreased desire for the pre-fed food (specific satiety). After pre-feeding, jays could observe a human hiding food in a caching tray before being able to access that tray. The jays participated in three trials, each on a separate day. A baseline trial in which jays were pre-fed a maintenance diet (MD) revealed an average preference for pilfering food A over food B (see raw data in Table S1 in the Supplemental Information). A direct comparison between the two test trials (jays pre-fed food A or food B) showed that this preference was influenced by the observers’ specific satiety: the preference for pilfering food A over food B relative to the baseline was smaller after jays had been pre-fed food A than after they had been pre-fed food B (n = 16, permutation test, Z = –2.61, p < 0.001, Cohen’s d = 0.833, Figure 1A). Thus, observers pilfer according to their current desire such that it would be beneficial for cachers to cache less of the food that an observer is most motivated to pilfer.

In the caching experiment, cachers and observers were tested in adjacent compartments. In the seen condition, cachers first witnessed observers being pre-fed a particular food and could subsequently cache both test foods in a caching tray. A baseline trial (observer pre-fed MD) revealed an average preference for caching food A over food B (Table S2). A direct comparison between the two test trials (observer pre-fed food A or food B) showed that this preference was influenced by the observer’s specific satiety: the preference for caching food A over food B relative to the baseline was larger after the observer had been pre-fed food A than after the observer had been pre-fed food B (n = 16, Z = 1.895, p = 0.006, Cohen’s d = –0.521, Figure 1B). Thus, the cachers protected their caches by selectively caching food that the observer currently did not desire.

In the unseen condition of the caching experiment, we investigated what information led cachers to alter their caching behaviour. The procedure was identical to before, except that cachers did not see what the observer had been pre-fed. Here too, cachers protected their caches by selectively caching food that the observer currently did not desire: the preference for caching food A over food B relative to the baseline was larger after the observer had been pre-fed food A than after the observer had been pre-fed food B (n = 16, Z = 2.329, p = 0.003, Cohen’s d = –0.693, Figure 1C). The caching pattern did not differ between the unseen and seen conditions (n = 16, Z = –0.731, p = 0.255, Cohen’s d = 0.180). Thus, cachers did not need to see what the observer ate to satiety to employ this particular cache-protection strategy. Instead, cachers might have responded to the observer’s behaviour during the caching event.

These findings have three implications. Firstly, cachers decrease cache loss not only through limiting the observer’s sensory access to the caching event, but also through preferentially caching items currently not desired by the observer. Secondly, cache-protection strategies found in previous studies have been interpreted as being based on the cachers’ ability to attribute perspective and knowledge-states to the observer [2]; in contrast, the current study highlights that the evolution of a flexible cache-protection strategy might not necessitate a highly complex cognitive process like state attribution. Finally, the cachers’ reliance on the observer’s behaviour during the caching event contrasts with a recent finding, according to which the male jays’ ability to adjust their food-sharing behaviour to their female’s current motivational state [10] might be based on desire-state attribution. This difference suggests that — depending on the context — behaviours that are conceptually similar might be subserved by different cognitive processes. These different cognitive processes might reflect differences in the duration and quality of social interactions across the different contexts. The cooperative context of courtship might allow prolonged interactions between mates and thus provide the opportunity to infer desire-states. In contrast, in the competitive context of caching, cachers might see competing conspecifics only briefly, such that it might make adaptive sense that a capacity to ‘read’ the observer’s behaviour during the caching event itself should evolve.

## Supplemental Information

Document S1. Supplemental Experimental Procedures and Two Tables
