# Supplementary material for: Current desires of conspecific observers affect cache-protection strategies in California scrub-jays and Eurasian jays
Source: Curr Biol. 2017 Jan 23;27(2):R51–3. doi: 10.1016/j.cub.2016.11.020 (PMC5266788; doi:10.1016/j.cub.2016.11.020)
Supplement: Document S1. Supplemental Experimental Procedures and Two Tables [file mmc1.pdf]

Supplemental Information: Current desires of conspecific observers affect cache protection strategies in California scrub-jays and Eurasian jays

Ljerka Ostojić, Edward W. Legg, Katharina F. Brecht, Florian Lange, Chantal Deininger, Michael Mendl and Nicola S. Clayton

Supplemental Experimental Procedure

Subjects

Ten California scrub jays (4 female, 6 male) and six Eurasian jays (3 female, 3 male) participated in the pilfering experiment, and nine California scrub jays (4 female, 5 male) and seven Eurasian jays (2 female, 5 male) participated in the caching experiment. California scrub jays were housed in pairs in indoor mesh cages and tested in cages of 1 x 1 x 1m. Eurasian jays were housed in large outdoor aviaries (20 x 6 x 3 m) and tested in indoor testing compartments (1 x 2 x 2 m). Outside of testing all jays had ad libitum access to their maintenance diet consisting of a mix of dog biscuits, seeds, nuts, cheese, vegetables and fruits. To ensure that the jays were mildly hungry and therefore motivated to pilfer or cache during the experiments, the maintenance diet was removed approximately 2 hours prior to testing. Jays had ad libitum access to water at all times and received different nuts as treats during testing. The experiments were approved by the University of Cambridge Ethics Review Committee.

Pilfering Experiment

Jays first experienced a pre-feeding phase, in which they had access to either a handful of maintenance diet (MD – baseline), 50 pieces of food A or 50 pieces of food B. Due to individual preferences, different types of nuts served as test foods for different jays (see Table S1). In the two test trials (pre-fed food A and food B), the experimenter also put a bowl containing the other type of food outside of the jays' cage/compartment to control for simple visual and olfactory cues. The jays were given the pre-feeding food in cage/compartment 1 in colour unique bowls to facilitate the distinction of the different foods. During this 15-minute long pre-feeding phase jays could either eat or cache the food inside cage/compartment 1. At the end of the pre-feeding phase the experimenter removed

the bowl and any remaining food. The experimenter then positioned a caching tray (ice cube tray with 4x7 pots for the scrub jays and a seedling tray with 5x5 pots for the Eurasian jays) outside cage/compartment 1 and hid 8 pieces of food A and 8 pieces of food B in a pre-determined sequence (order pseudo-randomised such that one type of food was not hidden more than twice in a row) and into pre-determined locations (pseudo-randomised such that no pot contained more than one nut). Using the corvids' propensity to pilfer human made caches [S1] allowed us to use human 'cachers' to control for number of items cached, time spent manipulating and caching each item as well as the probability that a location would contain a cache. Subsequently, the tray was positioned with the same orientation into cage/compartment 2. The experimenter opened a window connecting the cages/compartments 1 and 2 and allowed the jay to enter cage/compartment 2, after which the experimenter again closed the window. During the following pilfering phase, the jay could pilfer the nuts from the caching tray. After 15 minutes, the experimenter returned and removed the tray. The experimenter released the jay into its home cage or the outside aviary and recorded the number and locations of the remaining nuts in the tray. These data were then used to calculate which nuts were removed or re-cached into a different location by the jays and which remained in the original location.

All jays first received the baseline trial, in which they were pre-fed MD. The order of the subsequent test trials (pre-fed food A or B) was counterbalanced across jays. The baseline was conducted to account for individual variation in the general preference for pilfering foods A and B. California scrub jays received three trials for each of the test foods (thus, a total of 1 baseline trial plus 6 test trials) whilst Eurasian jays received one trial for each of the test foods (thus, a total of 1 baseline trial plus 2 test trials, each on a separate day). Unlike the Eurasian jays, which have experienced specific satiety in a number of different

experiments in the previous years [S2,S3], the scrub jays had not been tested on specific satiety for a relatively long period of time. Thus, they received three trials per test food to facilitate the development of specific satiety for the test foods.

Caching Experiment

Jays were tested with a second conspecific acting as an observer in an adjacent cage/compartment. During the pre-feeding phase, the future cacher was located in cage/compartment 2, whilst the future observer was located in cage/compartment 3. During the 15-minute long pre-feeding phase the future observer received either a handful of MD (baseline) or 50 nuts of type A or B (test trials). The future cacher received a handful of MD in all trial types such that its desire was neutral towards the test foods and constant across all trials. In the two test trials (future observer pre-fed food A and food B), the experimenter also put a bowl containing the other type of food outside of the future observer's cage/compartment to control for simple visual and olfactory cues. In the seen condition, the future cacher could see the future observer during the pre-feeding phase through a transparent Perspex window. The pre-feeding food was given to the future observer in colour unique bowls to facilitate distinction of the different foods. In the unseen condition, the Perspex window was covered with opaque sheeting such that the future cacher could not see the future observer during pre-feeding. In addition, the bowls used in the unseen condition were not colour unique and covered by a lid whenever they were in view of the future cacher. During the 15-minute long pre-feeding phase, the jays could either eat or cache the food. At the end of the pre-feeding phase the experimenter removed the bowls with the remaining food (if any).

At the beginning of the caching phase, the experimenter positioned a caching tray (ice cube tray with 4x7 pots for the scrub jays and a seedling tray with 5x5 pots for the Eurasian jays) into cage/compartment 1, opened up the window between cages/compartments 1 and 2 and allowed the cacher to access cage/compartment 1, after which the window was put back in place. Next, the experimenter opened the window between cages/compartment 2 and 3 and allowed the observer to access cage/compartment 2. This procedure ensured that the observer did not have access to the pre-feeding food that it might have cached during pre-feeding during the test phase of the experiment. Subsequently, the experimenter placed two colour unique bowls into cage/compartment 1, equidistant from the caching tray: one containing 50 pieces of food A, the other containing 50 pieces of food B. The cacher was then given 15 minutes in which it could cache the foods in the caching tray, after which the experimenter released both jays into their home cages/aviary and recorded the caches made.

To maintain the jays' motivation to cache, the cacher was given the opportunity to retrieve any caches made on the following day. In each condition, all jays started with the baseline, in which the partner jay was pre-fed MD, while the order of the test trials (future observer pre-fed food A and food B) was counterbalanced across jays. Thus, the jays were tested for 6 days in total per condition. The baseline was conducted to account for individual variation in the general preference for caching foods A and B. Additionally, the order in which the jays experienced the seen and unseen conditions was counterbalanced across jays. Those jays that were tested as both cachers and observers (see Table S2) always participated as cachers first.

Analysis

Data were live scored by KFB, FL and CD for the California scrub-jays and EWL and LO for the Eurasian jays. Following the analysis of Ostojić et al. (2014) [S2], for each trial, we calculated the number of items of food A minus the number of items of food B pilfered or cached (food A – food B). The two species did not differ in their performance in any of the experiments such that their data were pooled for all analyses and graphs. Graphs show the difference between these values in a test trial (pre-fed food A or food B) and the baseline (pre-fed MD): $[(\text{food A} - \text{food B})_{\text{pre-fed food A or food B}} - (\text{food A} - \text{food B})_{\text{pre-fed MD}}]$. This ensured that individual variation in the amount of food pilfered or cached as well as in general food preferences were taken into account. If the jays' specific satiety affected their pilfering behavior, in a direct comparison between the two test trials, the preference for pilfering food A over B relative to the baseline was expected to be lower when the jays were sated on food A than when they were sated on food B. If the observer's specific satiety affected the cacher's caching behavior, the preference for caching food A over food B relative to the baseline was expected to be larger when the observer was sated on food A than when it was sated on food B.

All analyses were planned contrasts, performed using exact permutation tests using the R package coin [S4]. Unless otherwise stated, all tests were one-tailed [S5]. Alpha was set at .05.

Supplemental References

S1. Bugnyar, T. and Heinrich, B. (2005). Ravens, *Corvus corax*, differentiate between knowledgeable and ignorant competitors. Proc. R. Soc. B. 272, 1641-1646.

S2. Ostojić, L., Legg, E.W., Shaw, R.C., Cheke, L.G., Mendl, M. and Clayton, N.S. (2014). Can male Eurasian jays disengage from their current desire to feed the female what she wants? Biol. Lett. 10: 20140042.

S3. Ostojić, L., Shaw, R.C., Cheke, L.G. and Clayton, N.S. (2013). Evidence suggesting that desire-state attribution may govern food sharing in Eurasian jays. Proc. Natl. Acad. Sci. USA 110, 4123-4128.

S4. Hothorn, T., Hornik, K., Wiel, M.A. van de and Zeileis, A. (2008). Implementing a class of permutation tests: the coin package. J. Stat. Softw. 28, 1-23.

S5. Cho, H. and Abe, S. (2013). Is two-tailed testing for directional research hypotheses tests legitimate? J. Bus. Res. 66, 1261-1266.

Table S1: Number of food items pilfered

						Test trial 1				Test trial 2				Trial 3			
				Pre-MD		Pre-A		Pre-B		Pre-A		Pre-B		Pre-A		Pre-B	
		Food A	Food B	A	B	A	B	A	B	A	B	A	B	A	B	A	B
California Scrub-jays	210	1/4 Pc	1/2 Pn	1	0	0.5	0	0.5	0	0	0	1	0	1	0	3	0
	202	1/4 Pc	1/2 Pn	0	0	0	0	0	1	0	0	0	0	0	0	1	1
	31	1/4 Pc	1/2 Pn	0	0	1	0	0.5	0	0	0	1	0	0	0	0	0
	108	1/4 Pc	1/2 Pn	0	1	0	0	0	0	0	0	0	0	0	0	1	0
	220	1/4 Pc	1/2 Pn	0	0	2	1	1	0	0	0	2	0	0	1	1	0
	229	1/4 Pc	1/2 Pn	0	0	0	0	0	0	0	0	1	0	0	0	0	0
	215	1/4 Pc	1/2 Pn	0	0	0	0	0	1	0	0	0	0	0	0	0	0
	224	1/4 Pc	1/2 Pn	1.5	0	0	0	1	0	0	0	0	0	0	0	0	0
	222	1/4 Pc	1/2 Pn	1	3	0	1	3.5	0	0	0	0	0	0	0	7	0
	203	1/2 Ps	1/4 Mn	0	1	0	2	0	0	0	0	0	0	0	0	0	0
Eurasian jays	Lima	1/2 Pn	1/4 Mn	5	4	0	2	4	3								
	Caracas	1/2 Pn	1/4 Mn	2	3	1	1	2	1								
	Rome	1/2 Pn	1/4 Mn	0	1	0	0	0.5	0								
	Washington	1/2 Pn	1/4 Mn	0	1	0	1	2	0								
	Dublin	1/2 Pn	1/4 Mn	1	0	0	0	2	0								
	Jerusalem	1/2 Pn	1/4 Mn	0	1	0	0	1	1								

The columns ‘Food A’ and ‘Food B’ denote the types of nuts that served as food A and food B for the individual jays as well as the proportion of the nut that was used as one piece of food: Pc = Pecans, Pn = Peanuts, Ps = Pistachios, Mn = Macadamia nuts. The column ‘Pre-MD’ denotes how many items of food A (column ‘A’) and food B (column ‘B’) have been pilfered after the jays had been pre-fed maintenance diet. The columns ‘Pre-A’ denote how many items of food A (columns ‘A’) and food B (columns ‘B’) have been pilfered after the jays had been pre-fed food A. The columns ‘Pre-B’ denote how many food items of food A (columns ‘A’) and food B (columns ‘B’) have been pilfered after the jays had been pre-fed food B. The maximum number of items that the jays could pilfer in one trial was 8 pieces of food A and 8 pieces of food B. To facilitate the development of specific satiety to the test foods (food A and food B), California scrub-jays were given three test trials. Eurasian jays were given one test trial only because these jays have recently participated in other experiments involving specific satiety.

Table S2: Number of food items cached in the seen and unseen conditions

					Seen						Unseen					
					Pre-MD		Pre-A		Pre-B		Pre-MD		Pre-A		Pre-B	
	Cacher	Observer	Food A	Food B	A	B	A	B	A	B	A	B	A	B	A	B
California Scrub-jays	202*	13	1/4 Pc	1/2 Pn	1	20	9	9	2	6	1	1	1	1	0	2
	210	202	1/4 Pc	1/2 Pn	10	20	1	14	0	7	15	1	6	9	2	7
	31*	13	1/4 Pc	1/2 Pn	12	0	7	0	7	1	16	0	16	0	6	1
	229*	108	1/4 Pc	1/2 Pn	10	0	2	1	5.5	0	1	1	5	0	4	0
	215*	229	1/4 Pc	1/2 Pn	1	0	1	0	0	6	0	0.5	2	2	1	0
	220	215	1/4 Pc	1/2 Pn	3	0	7	0	2	0	6	3	0	2	2	4
	224	202	1/4 Pc	1/2 Pn	13.5	16	19.5	3	11	5	2	30.5	4	5	2	8
	222	207	1/4 Pc	1/2 Pn	0	0	7	0	1	0	0	1	19	9	2	4
	203	31	1/2 Ps	1/4 Mn	27	1	40.5	0	0	2	50	2	28	0	8	0
Eurasian jays	Caracas	Quito	1/2 Pn	1/4 Mn	10	1	18	2	10	0	15	0	26	1	15.5	0
	Washington	Lima	1/2 Pn	1/4 Mn	1	3	1	1	2	10	2	1	0	0	3	1
	Hoy*	Pendleton	1/2 Pn	1/4 Mn	14	0	5	0	5	2	1	0	5	0	1	3
	Rome	Quito	1/2 Pn	1/4 Mn	8	1	13	0	13	6	9	0	3	0	0	4
	Dublin	Caracas	1/2 Pn	1/4 Mn	4.5	0	2	0	2	4	8	0	12	0	0	9
	Romero	Adlington	1/2 Pn	1/4 Mn	3	0	2	0	2	2	2.5	0	3	0	0	0
	Pendleton*	Hoy	1/2 Pn	1/4 Mn	3	0	3	0	3	2	4	0	2	0	0	3

The columns 'Cacher' and 'Observer' denote which jays participated in the caching experiment. * denotes jays that were used in both roles, in which case the jay always first participated as the cacher. The columns 'Food A' and 'Food B' denote the types of nuts that served as food A and food B for the individual jays as well as the proportion of the nut that was used as one piece of food: Pc = Pecans, Pn = Peanuts, Ps = Pistachios, Mn = Macadamia nuts. The column 'Pre-MD' denotes how many items of food A (column 'A') and food B (column 'B') have been cached by the cacher after the observer had been pre-fed maintenance diet. The columns 'Pre-A' denote how many items of food A (columns 'A') and food B (columns 'B') have been cached by the cacher after the observer had been pre-fed food A. The columns 'Pre-B' denote how many food items of food A (columns 'A') and food B (columns 'B') have been cached by the cacher after the observer had been pre-fed food B. The maximum number of items that the jays could cache in one trial was 50 pieces of food A and 50 pieces of food B.

Author Contribution:

Conceptualization, L.O.; Methodology, L.O., E.W.L., F.L. and K.F.B.; Investigation, L.O., E.W.L., F.L., K.F.B. and C.D.; Writing – Original Draft, L.O.; Writing – Review & Editing, L.O., E.W.L., F.L., K.F.B., C.D., M.M. and N.S.C.; Funding Acquisition: N.S.C., M.M. and E.W.L.; Resources: N.S.C.
